# Trace metals and nutrient analysis of marine fish species from the Gwadar coast

**DOI:** 10.1038/s41598-024-57335-0

**Published:** 2024-03-19

**Authors:** Masooma Khawar, Zubia Masood, Habib Ul Hasan, Wali Khan, Patricio R. De los Ríos-Escalante, Mashael Abdullah Aldamigh, Noorah Saleh Al-Sowayan, Wajeeha Razzaq, Tawseef Khan, Mourad Ben Said

**Affiliations:** 1 Department of Zoology, Sardar Bahadur Khan Women’s University, Quetta, Balochistan Pakistan; 2 Department of Zoology, University of Karachi, Karachi, Pakistan; 3 Fisheries Development Board, Ministry of National Food Security and Research, Karachi, Pakistan; 4 Department of Zoology, University of Malakand, Lower Dir, Khyber Pakhtunkhwa Pakistan; 5 Facultad de Recursos Naturales, Departamento de Ciencias Biológicas y Químicas Casilla, Universidad Católica de Temuco, 15-D Temuco, Chile; 6 Department of Biology, College of Science in Zulfi, Majmaah University, Al Majmaah, 11952 Saudi Arabia; 7 Department of Biology, College of Science, Qassim University, Buraidah, Saudi Arabia; 8 Department of Zoology, Abdul Wali Khan University, Mardan, Pakistan; 9 Laboratory of Microbiology, National School of Veterinary Medicine of Sidi Thabet, University of Manouba, 2010 Manouba, Tunisia; 10 Department of Basic Sciences, Higher Institute of Biotechnology of Sidi Thabet, University of Manouba, 2010 Manouba, Tunisia

**Keywords:** Marine fishes, High-quality proteins, Lipids, Trace metals, Nutrient analysis, Sustainable fishing practices, Gwadar coast, Zoology, Ecology, Ocean sciences

## Abstract

Trace metals are naturally occurring metals found in very small concentrations in the environment. In the context of fish flesh, metals such as copper, calcium, potassium, sodium, zinc, iron, and manganese are absorbed by fish and play vital roles in various physiological functions. However, if these metals exceed the recommended limits set by WHO/FAO, they are termed 'toxic metals' due to their harmful impacts on both the fish and its consumers. Therefore, the present study aims to analyze the levels of protein, lipids, and certain metals—Aluminum (Al), Sodium (Na), Zinc (Zn), Titanium (Ti), Iron (Fe), Copper (Cu), Potassium (K), and Calcium (Ca) in three commercially important marine fishes i.e. *Rastrelliger kanagurta, Sardinella abella*, and *Otolithes ruber*. The study also aims to assess their potential impact on human health. The macro-Kjeldhal method and Soxhlet apparatus were used to estimate protein and lipid contents, while atomic absorption spectroscopy (AAS) was used to estimate trace metals found in fishes. The study found that these fish species are valuable sources of protein, lipids, and certain essential minerals. The protein content (CP) in these three species ranged from 63.35 to 86.57%, while lipid content was from 21.05 to 23.86%. The overall results of the trace metal concentrations analyzed in the present study revealed that Aluminum (Al), Sodium (Na), Zinc (Zn), Titanium (Ti), Copper (Cu), Potassium (K), and Calcium (Ca) were found in low concentration or traces and also within suitable ranges as set by WHO/FAO. However, Iron (Fe) was absent in all three species. Moreover, both copper and potassium were found in all three species, while Zinc was present in *Rastrelliger kanagurta* and *Sardinella abella*, calcium in *Sardinella abella*, and sodium in *Otolithes ruber* only. Titanium was recorded for the first time in *S. abella*. However, the total health risk assessment associated with these fish food consumption was measured by THQ and TTHQ and found to be less than 1, which shows no potential risk related to trace metals found in these fishes on human health upon their consumption. In conclusion, these commercially important marine fish species were found valuable sources of protein, lipids, and essential trace minerals that are necessary for human health. Thus, the current study provides useful information for the local population to make informed decisions about their daily diets and highlights the importance of sustainable fishing practices to maintain these valuable marine resources by periodical monitoring of their ecosystem.

## Introduction

Fish is a valuable and affordable source of essential nutrients for humans, including protein, phospholipids, polyunsaturated fatty acids, certain minerals, vitamins, and Omega-3 fatty acids. These nutritional components support various biological processes in humans^1^. The nutritional content of fish depends on its diet. With the global population growing, the demand for fish, known for its high nutritional value, has increased^2^. Consuming fish has been linked to reduced risk of heart diseases, healing of wounds, breast and colon cancer, Alzheimer's disease, and improved immunological system^3^. Therefore, nutritionists widely recommend fish in our daily diets^4^. However, deficiencies in dietary protein and certain minerals found in fish in our daily diet have been identified as contributing factors to various health issues affecting approximately 2 billion people globally^5^. Certain fish species, especially cold-water varieties like cod, salmon, mackerel, herring, and sardines, contain unique oils that can reduce blood clotting tendencies, lower cholesterol levels, and are used to address deficiencies in vitamins A and D in our diet^6^.

Trace metals refer to minerals present in living tissues in smaller amounts. Trace metals are persistent and non-biodegradable and can be categorized as; (1) Essential trace metals that have important biological functions in living organisms; and (2) Non-essential metals that do not serve any known biological function. Iron, calcium, potassium, manganese, magnesium, zinc, sodium, phosphorus, cobalt, fluoride, copper, iodine, chromium, and selenium are essential nutritional trace elements required for the human body to function properly. Their primary function lies in catalyzing enzyme reactions^7^. Examples of non-essential trace metals include aluminum, nickel, cadmium, lead, mercury, and chromium^8^, which are commonly found in the aquatic environment as a result of atmospheric deposition, anthropogenic activities, and corrosion, leading to various health hazards for aquatic biota and can decline the levels of essential trace metals^9,10^. Additionally, another significant metalloid that bio-accumulates in fish is "Arsenic." It is extensively utilized in the manufacturing of metal alloys, microelectronics, glassware, wood preservatives, and veterinary drugs. Arsenic is transferred to the human population through the aquatic food chain, leading to the occurrence of numerous autoimmune and inflammatory diseases in countries such as China, Bangladesh, India, Vietnam, and the USA^11^.

The oceanic crust is primarily composed of a type of rock called basalt, which is rich in various mineral resources including i.e., iron, magnesium, calcium, aluminum, zinc, sodium, gold, and silver. However, the deposition of these minerals in the ocean can vary depending on their age and surrounding rocks. Fish found in the ocean can indeed accumulate these minerals in the form of trace metals from their surrounding aquatic habitat as well as from the organisms that they eat. These trace elements can also be directly absorbed by fish from the surrounding water through their gills and skin, which later accumulate especially in certain body tissues like the liver, kidneys, and muscles. The rate and extent of bioaccumulation of these metals depend on factors such as the fish's species, size, age, and diet, as well as the concentration of metals in the environment. The bioaccumulation of these metals in fish can raise concerns for both human and ecosystem health. Consuming fish with high levels of certain trace metals, such as mercury, lead, and copper can pose health risks to humans, particularly pregnant women and children. Therefore, it's important to monitor and regulate the levels of these metal contaminants in fish populations by reducing aquatic pollution and controlling the release of trace metals into marine environments are highly essential for minimizing these health risks in fish consumers^7,12^.

Fish meat also contains certain trace metals including i.e., zinc, copper, potassium, phosphorus, chlorine, iron, calcium, selenium, manganese, magnesium, chromium, cobalt, iodine, and fluoride, which are present in very small amounts. These trace metals play various roles in the physiology of fish and can also have implications for human nutrition when consuming fish^13^. The main functions of some of these trace elements in the human body, as outlined by FAO^14^, can be defined as follows; for instance, both iron and copper are involved in oxidation–reduction reactions crucial for energy metabolism. Iron, in particular, is indispensable, serving as a vital component of hemoglobin and myoglobin, facilitating the transport of oxygen in both the bloodstream and muscles^7^. **Iron (Fe)** is essential for the formation of hemoglobin in fish, just as it is in humans; **Zinc (Zn)** is important for various enzymatic reactions and metabolic processes, and also contributes to the functioning of the fish's immune system; **Copper (Cu)** is essential element involved in several biological processes, including oxygen transport, pigment formation, and enzyme function; **Potassium (K)** helps maintain acid–base balance within cells, is necessary for muscle function, and contributes to protein, glycogen, and glucose metabolism; **Calcium (Ca)** is essential for forming cartilage, bones, blood clotting, and regulating nerve impulses. It also aids in vitamin B12 absorption; **Sodium (Na)** is an essential element in fish physiology and is involved in maintaining osmotic balance, nerve impulse transmission, and muscle contractions. Sodium plays a critical role in the regulation of osmotic pressure and helps fish adapt to changes in salinity in their surroundings. **Aluminum (Al)** and **titanium (Ti)** are non-essential trace elements that can have varying roles and interactions in the physiology of fish. Aluminum (Al) is generally considered a non-essential element for fish. Fish can be exposed to aluminum through natural processes, such as the dissolution of aluminum compounds in water or sediment. High levels of aluminum in aquatic environments can be toxic to fish and potentially affect their gill function, respiration, and overall health. Titanium (Ti) is not known to have a specific role in fish physiology, and it is not considered an essential element for fish. Titanium is a naturally occurring element that is mainly used in the production of cosmetics, ceramics, silicon fibers, plastic fibers, and other chemicals in industries. Fish may come into contact with trace amounts of titanium discharge from these industries into their aquatic environment. Titanium is generally considered to be of low toxicity to aquatic organisms, including fish, at typical environmental concentrations. Fish is a valuable source of essential nutrients such as proteins, vitamins, lipids, carbohydrates, and certain essential trace minerals for humans. However, the consumption of fish sometimes can also introduce non-essential trace metals or toxic metals into the human body, which can have harmful effects on human health and contribute to the development of certain diseases^4^.

In Mediterranean countries, populations whose diets heavily rely on seafood are particularly exposed to metal intake^12^. Essential nutritional elements including Calcium, Sodium, Potassium, Phosphorus, Iron, and Chloride are crucial for maintaining human health. Deficiencies in any of these elements can lead to various disorders such as impaired blood clotting, anemia, bone problems, osteoporosis, growth disorders, and premenopausal and genetic disorders^15^. Therefore, it is essential to assess the protein, lipid, and mineral content of edible fish species to determine their nutritional value and suitability for meeting human dietary requirements, both from a biological and commercial perspective^16^.

In light of these considerations, this study aims to evaluate the trace metals and nutrient quality of three commercially significant edible fish species: white sardine (*Sardinella abella*), Indian mackerel (*Rastrelliger kanagurta*), and tiger mouth croaker (*Otolithes ruber*).

## Materials and methods

### Sampling area

**Figure 1:**
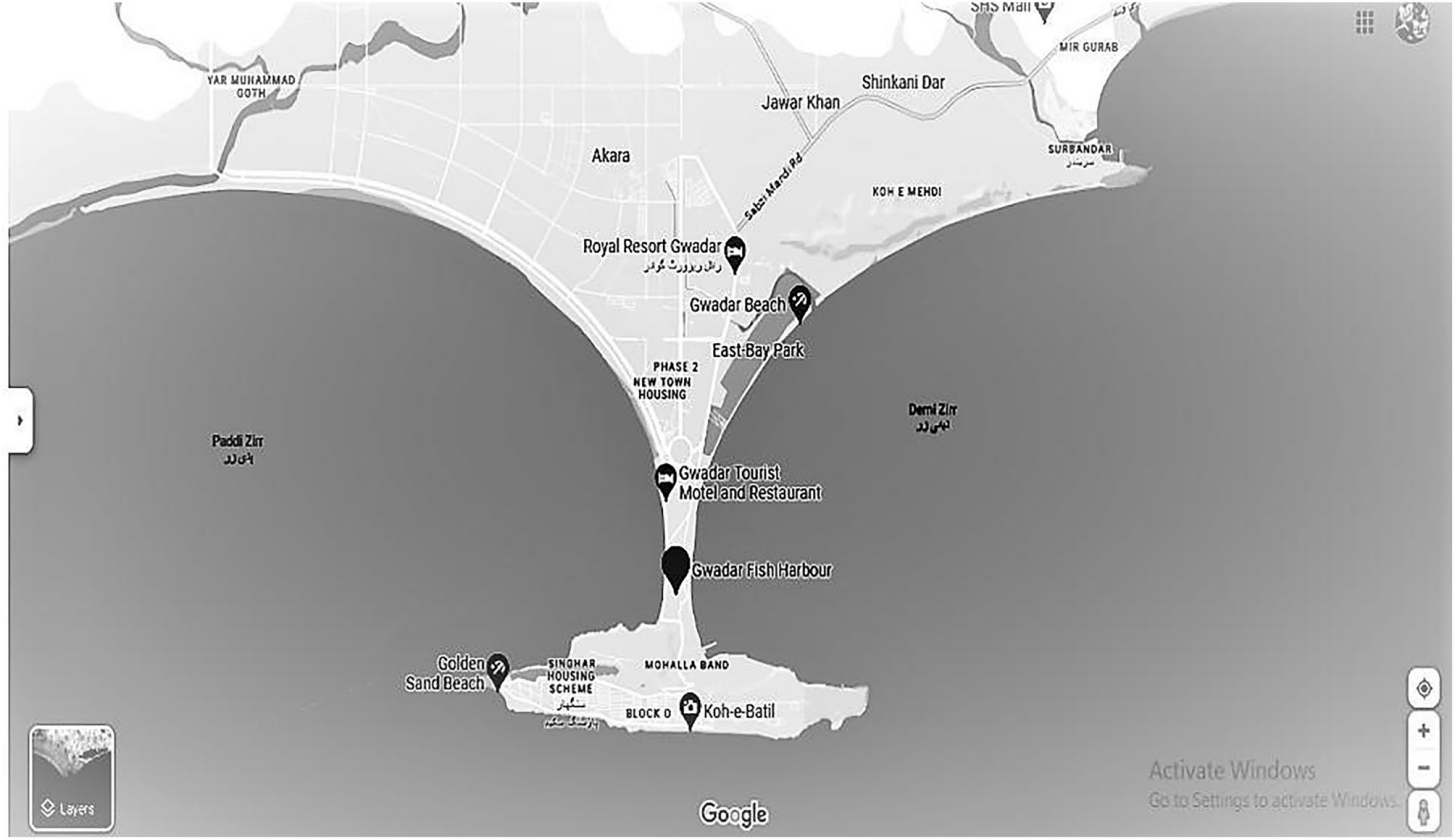
Google map shows the Fish Harbour of Gwadar Coast.

The study area is located in Balochistan, which encompasses a significant portion of the Arabian Sea coastline, including the collaborative project with China in Gwadar. Gwadar, a city in Balochistan, is situated at the beginning of the Persian Gulf at coordinates 25°07′35''N 62°19′21''E (Fig. 1). It covers a total area of 15,216 square kilometers and ranks as the 9th largest district in Balochistan Province. In the Balochi language, the term "Gwadar" means "gateway of winds"^17^.

### Approval of ethical review committee

Fishes used for the present study were fresh but lifeless. However, all procedure used during this study follow the guidelines of relevant standard methods. The study protocol and the ethics of this work have been approved by the Ethical Committee (Approval code: F14/MPhil/Zool-15 dated 10th September 2015) of the Sardar Bahadur Khan Women's University and confirmed that all methods were carried out in accordance with relevant guidelines and regulations. It is also confirmed all methods are reported in accordance with ARRIVE guidelines (https://arriveguidelines.org).

### Justification of animal use

Fish are commonly used in trace metal analysis research for several reasons including i.e., fish are excellent bioindicators of environmental contamination, including trace metals. They live in aquatic environments and accumulate metals from water, sediment, and the food chain. Therefore, analysis of trace metal concentrations in fish tissues can provide insights into the level of contamination in their aquatic habitat. Fish are an important part of the human diet, and the accumulation of certain trace metals in fish tissues can produce health risks in human body through the consumption of contaminated seafoods. Studying the trace metal levels in fish is important for assessing potential risks to human health and establishing guidelines for safe seafood consumption. Therefore, monitoring the trace metal levels in fish can help in assessing the overall health of aquatic ecosystems. Changes in metal concentrations may indicate pollution or alterations in the environment. This information is crucial for understanding the impact of human activities on aquatic ecosystems and for implementing conservation and remediation measures.

### Sample size

**Figure 2:**
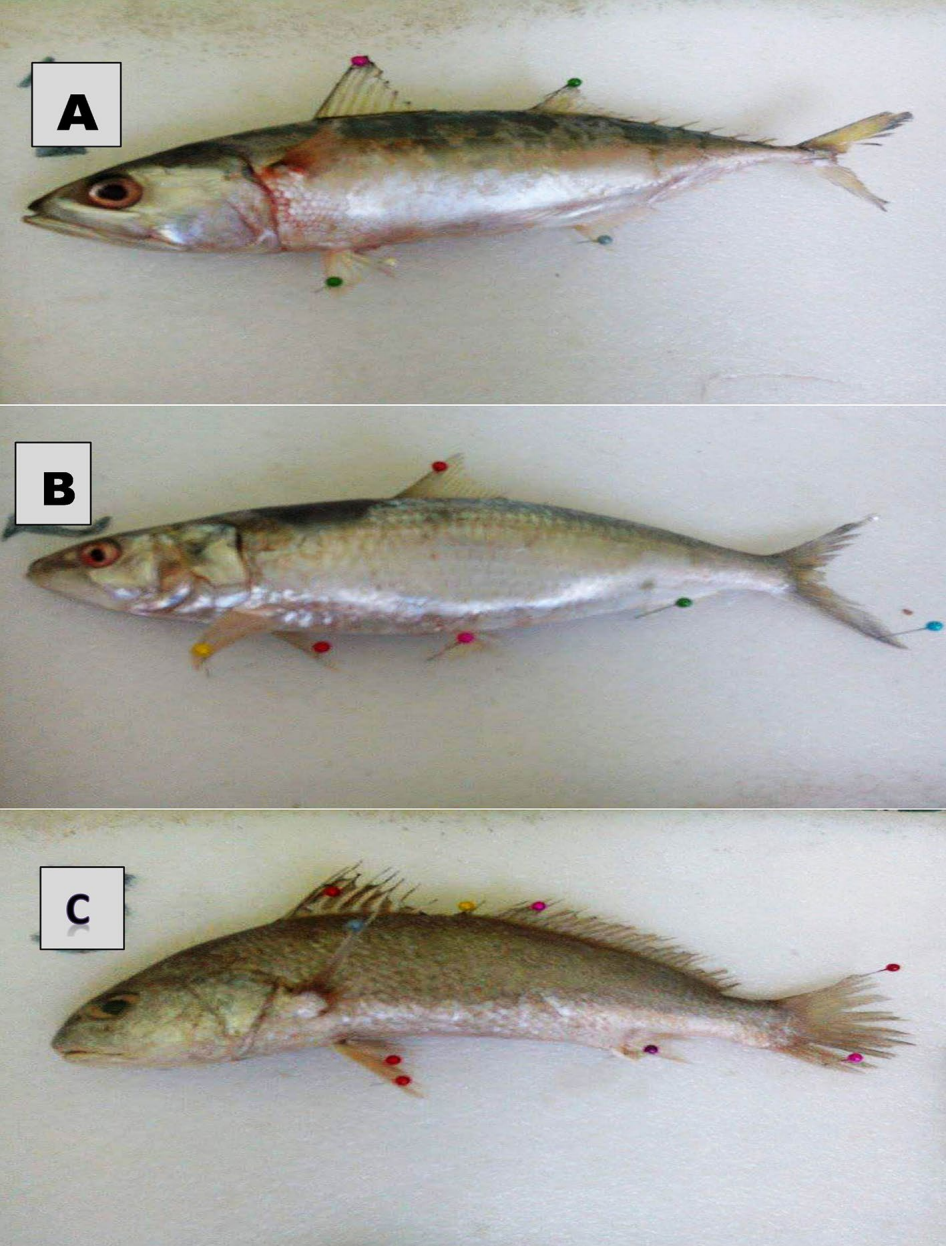
Fish samples of *Sardinella abella* (**A**), *Rastrelliger kanagurta* (**B**)*,* and *Otolithes ruber* (**C**).

A total of 100 samples were collected from three selected fish species. The samples consisted of white sardine (*Sardinella abella*, n = 30, mean total length (TL) = 21.2 ± 0.89 cm, weight (wt) = 111.4 ± 0.83 g) from the Clupeidae family, Indian mackerel (*Rastrelliger kanagurta*, n = 40, mean TL = 22.0 ± 0.85 cm, wt = 120.8 ± 1.68 g) from the Scombridae family, and tiger mouth croaker (*Otolithes ruber*, n = 30, mean TL = 21.6 ± 0.59 cm, wt = 150.6 ± 1.21 g) from the Sciaenidae family. The samples were collected from the landings at Gwadar fish harbor between January and December 2021 (Fig. 1). Fresh samples were transported to the laboratory for analysis of crude protein, lipids, and metal contents in the muscle tissues of these selected fish species (Fig. 2).

### Sample preparation for trace metals and nutrient analysis in fishes

The collected fish samples were placed in plastic bags and stored in deep freezers at the Fisheries Laboratory of the Zoology Department at Sardar Bahadur Khan Women's University, Quetta. The samples were further prepared for nutritional profile analysis by drying them in an oven at 60 °C for 6 h until no moisture remained. Subsequently, the dried samples were crushed into a powder form.

#### Analysis of crude protein contents

The determination of crude protein contents (CP) in the muscle tissues was conducted using the Macro-Kjeldahl method. This method is used to estimate nitrogen by digestion of sample in strong acid. This process is carried out in three steps include (1) Digestion, (2) Distillation, (3) Titration by using a Kjeldhal Apparatus.

##### Reagents

Sulphuric acid (H_2_SO_4_) nitrogen free, Potassium sulphate (K_2_SO_4_) as a catalyst, NaOH solution (40% w/v), Boric acid (4% w/v), 0.1 N standardized solution of hydrochloric acid (HCl), indicator (methyl orange).

##### Procedure

Take 2 g of fish muscle sample into a digestion flask. Then, add a digestion mixture that usually consists of 20 ml concentrated sulfuric acid (H_2_SO_4_) and a 7-g digestion catalyst (e.g., potassium sulfate). Set up a Kjeldahl digestion apparatus with a condenser to prevent the loss of volatile compounds. Heat the mixture gently at 400–420 °C to digest the sample until the solution turns light green because of the organic matter in the sample being oxidized, and nitrogen is converted to ammonium sulfate ((NH_4_)_2_SO_4_). After digestion, cool the sample in the flask and add 60 ml distilled water. Transfer the content into a distillation apparatus. Add a 30 ml solution of sodium hydroxide (NaOH), which converts ammonium sulfate into liberated ammonia gas. Collect this liberated ammonia gas into a 250 ml receiving flask containing 25 ml boric acid solution. The low pH of the solution in the receiving flask converts the ammonia gas into the ammonium ion, and simultaneously converts the boric acid to the borate ion. The nitrogen content is then estimated by titration of the collected ammonium borate with a standardized solution of hydrochloric acid (HCl), using 1 ml methyl orange as an indicator to signal the endpoint of this reaction. The volume of the acid solution used in the titration corresponds to the amount of ammonia released during the digestion.

Calculate the nitrogen content using the volume and normality of the acid solution as follows;

14 g of nitrogen contains one equivalent weight of NH_3_. The equivalent weight of NH_3_ is 17 g/eq. So, the percentage of nitrogen was determined by using formula mentioned below;

1 $$ \% {\text{ nitrogen content }} = {1}.{\text{4V }} \times {\text{ NW}}_{s} , $$

whereas; W_s_ = weight of fish muscle samples; N = normality of standard HCl; V = ml acid used in titration.

The percentage of protein nitrogen was calculated based on the Eq. (2) followed by Masood et al.^10^ as shown below:

2 $$ \% {\text{ Protein Nitrogen}}\, = \,\frac{{\left( {{\mathrm{b}} - {\mathrm{a}}} \right) \, \times \, 0.{1 } \times { 14}.00}}{{{\mathrm{W}}_{s} }} \times {1}00, $$

whereas; b = volume of 0.1N H_2_SO_4_ used in blank titration; a = 0.1N H_2_SO_4_ used in sample titration; W_s_ = weight of fish muscle samples; 1400 = atomic weight of Nitrogen.

Convert % protein nitrogen into % crude protein using a conversion factor, usually 6.25 for fish. Therefore, the percentage of crude protein was calculated based on using Eq. (2) followed by Masood et al.^10^ as shown below:

3 $$ \% {\text{ Crude}}\,{\text{Protein }} = \% {\text{ protein}}\,{\mathrm{Nitrogen}}\, \times \,{6}.{25}{\mathrm{.}} $$

#### Analysis of crude lipids

For lipid analysis, extraction was performed on the samples using petroleum ether. The percentage of crude lipid was determined using the Soxhlet apparatus, following the methods described by Hafiz et al.^18^.

##### Procedure

Assemble the Soxhlet apparatus with a 60 ml siphoning capacity and a condenser, ensuring that all joints are tight and well-sealed. Then place accurately weight 5 g of fish muscle sample into the cellulose extraction thimble. Dry this sample in an oven at 102 °C for 5 h. Next, insert this loaded thimble into the main Soxhlet chamber. Place a small amount of boiling chips into the accurately weighed, clean and dry 150 ml round-bottom flask, to prevent bumping during heating. Then add 90 ml of petroleum ether (extraction solvent) into this flask. The extraction process begins by heating the extraction solvent in the round-bottom flask until it boils. The solvent vaporizes, travels up the Soxhlet arm, and extracts lipids from the sample in the thimble. Continue the extraction cycles for 6 h until the solvent in the round-bottom flask becomes clear or nearly clear. This indicates that the lipids have been extracted from the sample. Place the flask in an oven at 60 to 80 °C for 1 to 2 h and dry the contents until a constant weight is obtained. Weigh the flask with the extracted crude lipids to determine the yield. Calculate the percentage of lipids in the original sample based on the weight of the extracted crude lipids.

The following formula was used to calculate the percentage of crude lipids as follows;

4 $$ \% {\text{ of crude lipids }} = \frac{{\left( {{\mathrm{W}}_{{2}} - {\mathrm{W}}_{{1}} } \right)}}{{{\mathrm{W}}_{{\mathrm{s}}} }} \times { 1}00. $$

While, W_2_ = Weight of flask with extracted lipids (g); W_1_ = weight of empty flask (g); W_s_ = Weight of dried fish muscle sample.

#### Analysis of trace metal contents in fishes

The levels of certain trace metals, including Aluminum (Al), Sodium (Na), Zinc (Zn), Titanium (Ti), Iron (Fe), Copper (Cu), Potassium (K), and Calcium (Ca), in the fish samples were determined using Atomic Absorption Spectroscopy (model Analyat 700 USA) as per standard protocols given by Din et al.^19^ with details of Intrumental parameters are given in Table 1. These results were presented as µg/g against each trace metal of this study. The determination of detection limits (DL) and quantification limits (QL) for various elements using Atomic Absorption Spectrophotometry (AAS) analyzed in present study. A series of standard solutions with known concentrations of the elements of interest were prepared. These standards should cover a range of concentrations that includes both the expected range of concentrations in your samples and concentrations near the anticipated DL and QL. Analyze the standard solutions using the AAS instrument and plot a calibration curve for each element. The calibration curve is a plot of the instrument response (signal intensity) versus the concentration of the analyte.

**Table 1:** Instrumental parameters for Atomic Absorption Spectroscopy (AAS) using Analyst 700 Metals.

Metals	Symbols	Wavelength (nm)	Flame type	Flame flow rate (L min^−1^)	Slit width (nm)
Aluminum	Al	309.3	Nitrous oxide-acetylene flame	6.0	0.2
Potassium	K	766.5	Air-acetylene flame	17.0	1.0
Copper	Cu	324.8	Oxygen-acetylene flame	2.0	0.5
Zinc	Zn	213.8	Nitrous oxide-acetylene flame	6.0	1.0
Calcium	Ca	422.7	Air-acetylene flame	17.0	0.2
Titanium	Ti	336.1	Nitrous oxide-acetylene flame	6.0	0.2
Sodium	Na	589.0	Air-acetylene flame	17.0	0.5
Iron	Fe	248.3	Oxygen-acetylene flame	2.0	0.2

The DL is typically calculated as three times the standard deviation of the blank signal divided by the slope of the calibration curve using the following equation of Kakar et al.^20^ as follows;

5 $$ {\mathrm{DL}} = {\text{ Xb1 }} + {\text{ 3Sb1}}, $$

whereas, Xb1 = average blank concentration, and Sb1 = standard deviation of the blank concentrations.

Likewise, QL is usually calculated as ten times the standard deviation of the blank signal divided by the slope of the calibration curve using the following equation Kakar et al.^20^ as follows;

6 $$ {\mathrm{QL}} = {\text{ Xb1 }} + { 1}0{\mathrm{Sb1}}. $$

Obtain the blank signal by measuring the signal (absorption intensity) in the absence of the analyte. This represents the baseline noise or interference from the sample matrix and reagents. The slope of the calibration curve represents the change in signal intensity per unit change in analyte concentration. It's a measure of the sensitivity of the method. Calculate the standard deviation of the blank signal from the signal readings obtained from the blank solutions. This quantifies the variability in the baseline signal. The methods used to determine the level of trace elements using AAS followed Nazeer et al.^7^. The accuracy of the analytical procedure of AAS was checked by the analysis of certified values from Standard Reference Material (SRM) 3233^21^ are mentioned in Tables 1 and 2.

**Table 2:** Shows Certified Values for Elements in SRM 3233 Mass Fraction (mg/kg), Wavelength in nm, measured values of each fish species, Detection limit (DL), Quantification limit (QL), % of Relative Standard Deviation (RSD) and Recovery.

Metals	Wavelength (nm)	*Certified values (mg/kg)	Measured average values of R. kanagurta (M1)	Measured average values of S. abella (M2)	Measured average values of O. rubber (M3)	Detection limits (DL) (µg/mL)	Quantification limit (QL) (ppm)	Relative Standard Deviation (%RSD)	% Recovery
Al	309.3	598.4 ± 7.1	0.66	ND	ND	0.1–1.0	0.01–1.0	1–5	99
K	766.5	3060.0 ± 140.0	2.675	1.94	2.24	1–10	0.1–10	1–4	90
Cu	324.8	275.2 ± 4.6	12.71	4.14	1.94	0.1–1.0	0.01–1.0	1–5	90
Zn	213.8	628.0 ± 16.0	6.64	2.96	ND	0.1–1.0	0.01–1.0	1–4	97
Ca	422.7	36,910.0 ± 920.0	ND	0.77	ND	1–10	0.01–1.0	1–3	98
Ti	336.1	NA	ND	0.45	ND	1–10	0.01–1.0	1–5	80
Na	589.0	6830.0 ± 120.0	ND	ND	0.94	1–10	0.1–10	1–3	98
Fe	248.3	766.0 ± 36.0	ND	ND	ND	0.1–1.0	0.01–1.0	1–5	99

*Standard Reference Material (SRM) 3233^21^.

*NA* not available, *ND* not detected.

##### Digestion of fish samples

For the dry method, the total collected fish samples of each species were divided into three replicates (10 for each group), and were designated as A1, B1, and C1 (*Rastrelliger kanagurta*), A2, B2, and C2 (*Sardinella abella*), and A3, B3, and C3 (*Otolithes ruber*). Then each replicate sample (group of 10 fishes) was oven-dried at a temperature of 180 °C for 2 h. After removing the complete moisture from them, they were taken out of the oven and converted into homogenized dry powder using a pestle and mortar.

##### Extraction of trace metals from fish samples

The powdered fish samples were placed in flasks contained 20 ml concentrated sulphuric acid (H_2_SO_4_) and heated on a hot plate. The temperature range for heating was set between 200 to 250 °C. This heating process continued until the solutions in the flasks became transparent and clear. During the heating process, brown fumes were observed initially. After these brown fumes, a dense white fumes appeared. The reaction involves the oxidation of organic matter (fish muscles) during acid digestion, and liberate sulfur dioxide gas (SO_2_) as one of the by-products. Thus, this change in fumes were used as an standard indication that the digestion process of sample was completed. Therefore, white fumes suggests that all the components in the samples had been digested. The digestion process was completed in a relatively short time frame, estimated to be between 2 to 4 h. After digestion, the samples were allowed to cool. Once cooled, they were diluted with Nano pure distilled water. The dilution was carried out to bring the samples to a suitable concentration for further analysis. Standard solutions prepared from stock standard solutions were used for the detection of trace metal compositions. These standard solutions likely contained known concentrations of the trace metals of interest. The diluted samples were compared to these standards to determine the concentration of trace metals in the fish samples using the methods followed by Nazeer et al.^7^.

### Calculation for health risk due to metal contamination

#### Estimated daily metal intakes (EDMI)

To evaluate the health risks associated with metal contamination in different fish species, the daily dose of metal intake was calculated using the following formula proposed by Kakar et al.^20^:

7 $$ {\text{EDMI }} = {\text{ FIR }} \times {\text{ C}}/{\mathrm{BW,}} $$

where FIR represents the fish ingestion rate (observed as 0.1424 kg/day in Balochistan province of Pakistan according to Kakar et al.^20^, C is the average metal concentration in fish samples (micrograms per gram of fish flesh on a dried weight basis), and BW is the average body weight of 52 kg for an adult human of the local population of Balochitan as suggested by Kakar et al.^20^.

#### Calculation of fish consumption data

The average fish consumption rate of 5.81 kg/capita/annum in the Balochistan human population was documented by the National Bureau of Statistics (Pakistan) and the Food and Agriculture Organization (FAO) surveys^22,23^.

While the Target hazard quotient (THQ) values of the trace elements for consumption by different marine fishes were used to evaluate the health risk assessment e.g., non-carcinogenic and carcinogenic risks of all these metals in three marine fish species eaten by the consumers at adult age as follows;

8 $$ {\text{THQ }} = \, \left( {{\text{EF }} \times {\text{ ED }} \times {\text{ FIR }} \times {\text{ C}}} \right)/\left( {{\text{RFD }} \times {\text{ BW }} \times {\text{ AT}}} \right). $$

whereas, C = the concentration of trace metal; FIR = the ingestion of each fish species (0.1424 kg/person/day); ED = duration of exposure (for adults: 70 years); EF = the exposure frequency (365 days/year) for adults; BW is body weight (for adult = average 52 kg); AT = the average time lifespan (10,950 days); RfD is oral reference dose (EPA, FAO/WHO).

Using all the trace element analyses from each species of fish samples, the sum of THQ of individual chemical elements (Eq. (9)) was calculated as the total target hazard quotient (TTHQ) as follows;

9 $$ {\text{TTHQ }} = {\text{ THQ}}^{{{\mathrm{Al}}}} + {\text{ THQ}}^{{{\mathrm{Na}}}} + {\text{ THQ}}^{{{\mathrm{Zn}}}} + {\text{ THQ}}^{{{\mathrm{Ti}}}} + {\text{ THQ}}^{{{\mathrm{Fe}}}} + {\text{ THQ}}^{{{\mathrm{Cu}}}} + {\text{ THQ}}^{{\mathrm{K}}} + {\text{ THQ}}^{{{\mathrm{Ca}}}} . $$

If THQ or TTHQ was ≥ 1, consumers are at considerable health risk for these metals ingestion, but when THQ or TTHQ < 1, then is no non-carcinogenic health risk is associated with these fish consumptions.

### Statistical analysis of data

All statistical analyses, including ANOVA and Tukey's multiple comparison tests at a significance level of p < 0.05, were performed using MS Excel version 365 and Minitab Statistical Software version 17.0.

### Ethical statement

Fishes used for the present study were fresh but lifeless. However, all procedure used during this study follow the guidelines of relevant standard methods. The study protocol and the ethics of this work have been approved by the Ethical Committee (Approval code: F14/MPhil/Zool-15 dated 10th September 2015) of the Sardar Bahadur Khan Women's University and confirmed that all methods were carried out in accordance with relevant guidelines and regulations. It is also confirmed all methods are reported in accordance with ARRIVE guidelines (https://arriveguidelines.org).

## Results

The length and weight data of the studied fish species are presented in Table 3.

**Table 3:** Size and weight data of three marine fish species collected from Gwadar Coast from January to December 2021.

Family	Species	Number of samples (N)	Mean ± S.D of TL in (cm)	Length range (TL) (cm)	Mean ± S.D of Wt (g)	Weight (Wt) range (g)
Scombridae	*R.kanagura*	40	22.0 ± 0.85	20.0–23.8	120.8 ± 1.68	117.0–124.0
Clupeidae	*S. abella*	30	21.2 ± 0.89	19.8–23.5	111.4 ± 0.83	110.0–113.0
Sciaenidae	*O. ruber*	30	21.6 ± 0.59	20.5–22.7	150.6 ± 1.21	148.0–153.0

TL is total length in cm. and Wt is weight of fish in gram.

*S.D* standard deviation.

### Protein and lipid contents

**Figure 3:**
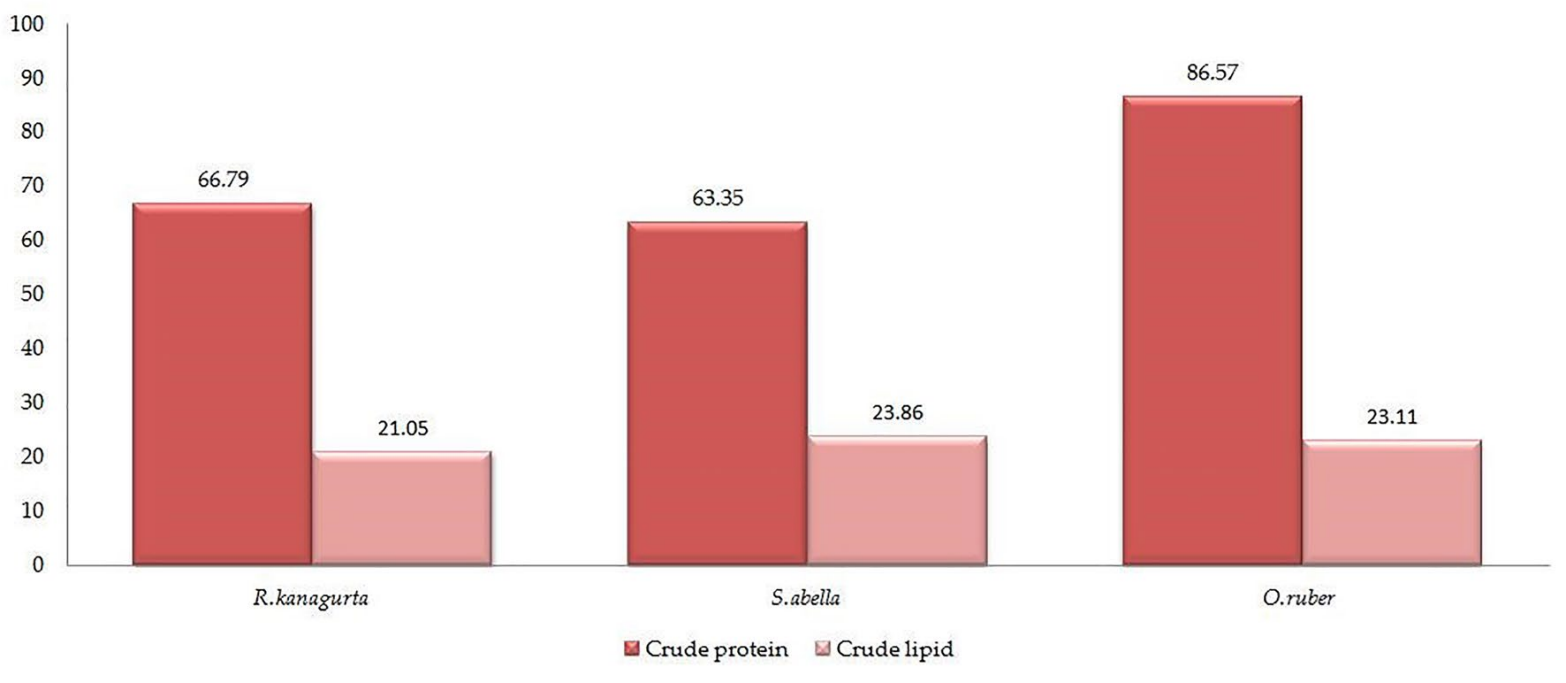
Crude protein (CP) and lipid contents in three edible marine fish species collected from Gwadar Coast.

The analysis of crude protein content (CP) revealed significant variations among the three fish species, ranging from 63.35% to 86.57%. *O. ruber* exhibited the highest CP value, while *S. abella* had the lowest. Regarding crude lipid content, slight differences were observed among the fish species, with values ranging from 21.05% to 23.86%. The descending order of crude lipid content was *S. abella* > *O. ruber* > *R. kanagurta*, as illustrated in Fig. 3.

### Trace metal composition in marine fishes

In the present study, we have identified six trace metals i.e., sodium (Na), zinc (Zn), iron (Fe), copper (Cu), potassium (K), and calcium (Ca) as essential trace metals, while aluminum (Al) and titanium (Ti) as non-essential trace elements in three fish species i.e., *R. kanagurta*. *Sardinella abella* and *Otolithes ruber* from Gwadar coast of Balochistan. In this study, the average concentration of eight trace metals in *Rastrelliger kanagurta, Sardinella abella* and *Otolithes ruber* was found within permissible limits of EPA/FAO/WHO (Tables 4, 5, 6). Moreover, the analysis of trace metal contents indicated the presence of potassium (K) and copper (Cu) in all three species. Aluminum (Al) was found exclusively in *Rastrelliger kanagurta*, while zinc (Zn) was detected in both *Rastrelliger kanagurta* and *Sardinella abella*. Calcium (Ca) was identified in *Sardinella abella* only, while sodium was detected only in *Otolithes ruber*. *Sardinella abella* also contained titanium (Ti), while iron (Fe) was absent in all three species (Tables 4, 5, 6). Notably, copper (Cu) exhibited the highest concentration among all the metals found in these species.

**Table 4:** Trace metal composition of *Rastrelliger kanagurta* with their permissible limits.

Metals	Symbols	Metal concentration (µg/g)			Mean (M1) ± S.D	EPA (Environmental protection Agency)/FAO/WHO permissible limits in µg/g (Kakar et al. 2020)
		Sample A1	Sample B1	Sample C1		
Aluminum	Al	ND	0.66	ND	0.66 ± 0.00	1.09
Potassium	K	ND	3.34	2.01	2.675 ± 0.94	8.78
Copper	Cu	ND	15.19	10.22	12.71 ± 3.51	30
Zinc	Zn	ND	7.60	5.68	6.64 ± 1.36	40
Calcium	Ca	ND	ND	ND	ND	75
Titanium	Ti	ND	ND	ND	ND	1500
Sodium	Na	ND	ND	ND	ND	29.4
Iron	Fe	ND	ND	ND	ND	100

*M1* mean, *S.D* Standard Deviation, *ND* Not detected, *NA* Not available.

**Table 5:** Trace metal composition of *Sardinella abella* with their permissible limits.

Metals	Symbols	Metal concentration (µg/g)			Mean (M2) ± S.D	EPA (Environmental protection Agency)/FAO/WHO permissible limits in µg/g^20^
		Sample A2	Sample B2	Sample C2		
Aluminum	Al	ND	ND	ND	ND	1.09
Potassium	K	2.52	1.36	ND	1.94 ± 0.8	8.78
Copper	Cu	6.87	3.44	2.11	4.14 ± 2.5	30
Zinc	Zn	4.49	ND	1.43	2.96 ± 2.2	40
Calcium	Ca	ND	ND	0.77	0.77 ± 0.00	75
Titanium	Ti	ND	ND	0.45	0.45 ± 0.00	1500
Sodium	Na	ND	ND	ND	ND	29.4
Iron	Fe	ND	ND	ND	ND	100

*M2* mean, *S.D* standard deviation, *ND* not detected, *NA* not available.

**Table 6:** Trace metal composition of *Otolithes ruber* with their permissible limits.

Metals	Symbols	Metal concentration (µg/g)			Mean (M3) ± S.D	EPA (Environmental protection Agency)/FAO/WHO permissible limits in µg/g^20^
		Sample A3	Sample B3	Sample C3		
Aluminum	Al	ND	ND	ND	ND	1.09
Potassium	K	3.10	2.91	0.70	2.24 ± 1.33	8.78
Copper	Cu	2.12	1.75	ND	1.94 ± 0.26	30
Zinc	Zn	ND	ND	ND	ND	40
Calcium	Ca	ND	ND	ND	ND	75
Titanium	Ti	ND	ND	ND	ND	1500
Sodium	Na	0.68	1.19	ND	0.94 ± 0.36	29.4
Iron	Fe	ND	ND	ND	ND	100

*M3* mean, *S.D* Standard Deviation, *ND* Not detected, *NA* Not available.

A two-sample t-test was conducted to compare the mineral compositions of the three replicate samples of each selected species, and only insignificant variations (P > 0.05) were observed among A vs. B, B vs. C, and C vs. D samples of *R. kanagurta*, *S. abella* and *O. ruber* (Table 7). Furthermore, ANOVA and Tukey's multiple comparison tests at a 95% confidence interval (CI) were performed to analyze the differences in mineral contents among the three selected species, and the overall results indicated insignificant variations (P > 0.05) (Tables 8 and 9).

**Table 7:** 2-sample t-test at 5% significance among the mineral composition in three samples of each selected species from Gwadar Coast.

*R. kanagura*				*S. abella*			*O. ruber*		
	95% CI (μ1–2)	T-test	p-value	95% CI (μ1–μ2)	T-test	p-value	95% CI (μ1–μ2)	T-test	P-value
A *vs.* B	0.0	0.0	0.0	− -1.06, 3.49	1.19	0.262*	− 1.31, 1.22	− 0.07	0.948*
B *vs.* C	− 3.95, 6.22	0.49	0.635*	− 1.17, 1.09	− 0.08	0.938*	− 0.13, 1.82	2.03	0.082*
A *vs.* C	0.0	0.0	0.0	− 1.04, 3.39	1.22	0.257*	− 0.22, 1.83	1.85	0.107*

95% CI for difference (μ1–μ2).

*Shows variations are insignificance when p > 0.05.

**Table 8:** One-Way ANOVA for analysis of variations between means of the trace metal compositions among the three different selected species collected from Gwadar Coast.

Fish species	Symbol (Means)	Mean ± S.D	95% CI	ANOVA at significance level α = 0.05 (when P < 0.05)					
				Source	DF	Adj SS	Adj MS	F-value	P-value
*R. kanagura*	M1	1.91 ± 3.1	(0.46, 3.35)	Factor	2	6.41	3.206	0.83	0.449*
*S. abella*	M2	1.01 ± 1.21	(− 0.425, 2.463)	Error	21	81.02	3.858		
*O. ruber*	M3	0.68 ± 0.85	(− 0.761, 2.128)	Total	23	87.43			

*CI* confidence interval.

*Shows insignificant variations when P > 0.05.

**Table 9:** Tukey Simultaneous tests and 95% Confidence interval (CI) for differences of means among the three selected species collected from the Gwadar Coast.

Difference of samples	Difference of means	SE of difference	95% CI	T-test	P-value
M2—M1	− 0.890	0.982	(− 3.362, 1.583)	− 0.91	0.643*
M3—M1	− 1.225	0.982	(− 3.697, 1.247)	− 1.25	0.440*
M3—M2	− 0.335	0.982	(− 2.808, 2.137)	− 0.34	0.938*

*Shows the p-value insignificant when P > 0.05; Individual confidence level = 95.0%.

### Estimated daily metal intake (EDMI) and recommended daily allowances(RDA) of trace metals

Table 10 shows the estimated daily metal intake (EDMI) and Recommended daily allowances (RDA) values in mg/person/day for the three fish species collected from the Gwadar coast (Tables 4, 5, 6).The overall results shows that the EDMI measured values of each trace metal analyzed in three fish species of Gwadar coast were found in the recommended range (RDA) except copper in *R. kanagurta*. Therefore, these species can fulfill the daily metal intake requirements of humans.

**Table 10:** Coverage of the estimated daily metal intake (EDMI) and recommended daily allowances (RDA) of metals in mg/day/person for consumers with average weight 52 kg.

Trace metals	Symbols	EDMI (mg/day/person)			RDA (mg/day)	References
		*R. kanagurta*	*S. abella*	*O. ruber*		
Aluminium	Al	1.81	ND	ND	6–14	FAO^24^; USEPA^25^
Potassium	K	7.33	5.31	0.00061	3400 to 4700	FAO^24^; USEPA^25^
Copper	Cu	3.48	1.13	0.0053	1–1.6	FAO^24^; USEPA^25^
Zinc	Zn	1.82	8.11	ND	8 to 11	FAO^24^; USEPA^25^
Calcium	Ca	ND	2.11	ND	1000 to 1300	FAO^24^; USEPA^25^
Titanum	Ti	ND	1.23	ND	1500	FAO^24^; USEPA^25^
Sodium	Na	ND	ND	0.0026	1500–2300	FAO^24^; USEPA^25^
Iron	Fe	ND	ND	ND	14; 8 to 18	FAO^24^; USEPA^25^

EDMI values in bold shows above RDA values.

### Human health risk assessments

In this study, the potential harm of trace metals in fish to humans through the consumption of food contaminated with them was evaluated by calculating the target hazard quotient (THQ) values (Table 11). If THQ is lower than 1, then such food eaten does not produce any acute adverse health effects. But in our present study, THQ values of eight trace elements in *Rastrelliger kanagurta, Sardinella abella* and *Otolith ruber* were less than one (THQ < 1). Hence these three fish species show no adverse human health effects on their consumption. While calculating the total health risk assessment for each fish species (TTHQ) related to their consumption, it was observed that all three species show TTHQ < 1, hence indicating that there was no potential health risk associated with these metal pollutants to their consumers.

**Table 11:** Human health risk assessment of trace metal composition in three marine fish species.

Trace metals	Symbols	Target hazard quotient (THQ)		
		THQ_Rk_	THQ_Sa_	THQ_Or_
Aluminum	THQ^Al^	0.004	ND	ND
Potassium	THQ^K^	0.004	0.0014	0.0016
Copper	THQ^Cu^	0.002	0.0009	0.0004
Zinc	THQ^Zn^	0.003	0.0005	ND
Calcium	THQ^Ca^	ND	0.0001	ND
Titanium	THQ^Ti^	ND	0.0000	ND
Sodium	THQ^Na^	ND	ND	0.0002
Iron	THQ^Fe^	ND	ND	ND
Total Target hazard quotient	TTHQ	0.010	0.003	0.002

*ND* Not detected, *THQ*_*Rk*_ Target hazard quotient of *Rastrelliger kanagurta*, *THQ*_*Sa*_ Target hazard quotient of *Sardinella abella*, *THQ*_*Or*_ Target hazard quotient of *Otolithes ruber* *THQ > 1.0 or **TTHQ > 1.0 = consumers at health risk for these metal ingestions.

## Discussion

### Protein and lipid contents

Fish is widely recognized as a valuable provider of essential nutrients, including high-quality proteins and lipids, which play a vital role in promoting human well-being. Therefore, the present study based on analyzing the nutritional content of commercially important marine fishes found along the Gwadar coasts of Pakistan revealed that selected marine fish species are valuable sources of protein, lipids, and essential minerals. Understanding the nutritional composition of fish species is crucial for assessing their potential as a valuable protein source for human consumption. Our study focuses on the findings related to the percentage of crude protein and lipid contents, as well as the factors influencing their variations among different fish species. Additionally, the presence of trace metals in these fish species is examined to evaluate potential health risks associated with metal contamination. Fish is highly valued in human diets due to its appealing flavor, rich protein content, and numerous nutritional benefits^26^. The percentage of crude protein in the present study indicates high protein contents with slight variations in CP values, which aligns with the findings of Masood et al.^10^. They also reported high crude protein content in four different mullet species from the Karachi coast of Pakistan. Similarly, Elagba et al.^27^ found high crude protein content ranging from 59.8% to 79.1% in five commercially important Nile fishes. Consistent with our study, Osibona et al.^28^ estimated a high protein content value in *Clarias garipinus*. The observed variations in crude protein content among fish species may be attributed to factors such as environmental changes, growth stages, and seasonal fluctuations, as suggested by Munshi et al.^6^.

Regarding lipid content, the present study indicates slight variations among the analyzed fish species, which is in agreement with Mnari-Bhouri et al.^29^, who estimated a similar range of lipid content in sea bass. Lipids are stored in both the muscles and liver of fish, and if there is a higher demand for lipid metabolism, additional lipids may be deposited in the fish's muscles. The variations in lipid content observed among different fish species could be attributed to environmental and biological factors that influence lipid metabolism and accumulation^30^. Fish lipid or fat content is influenced by environmental factors such as diet, age, sex, spawning, and food sources^31^. Omega-3 fatty acids, like Eicosapentaenoic acid (EPA) and Docosahexaenoic acid (DHA), commonly found in marine fish, offer significant health benefits. They can help prevent heart diseases, inflammatory diseases, allergies, breast and colon cancer, and various immunological disorders^32^.

### Trace metal composition in fishes

Balochistan is the largest province of Pakistan and is renowned for being the most mineral-rich region in the country. It is rich in various minerals, including indigenous deposits of iron, copper, gold, silver, lead, zinc, chromite, coal, gypsum, limestone (including marble varieties), and silica sand etc. Makran Coast of Balochistan contain three districts like Gwadar, Kech, and Panjgur. District Gwadar is a port city of Balochistan that contain a 620 km coastline along Arabian Sea. The mpst signifiacnt features of Gwadar Port is its deep sea warm water port.The region around Gwadar coast has been of interest for its potential mineral resources, such as, limestone, chromite, gypsum and other minerals particularly deposits on lands and Gwadar sea coast due to industrial and mining activities^33^. The accumulation of trace elements and metalloids in fish muscles depends on various factors, including habitat (whether the fish is a bottom dweller or surface feeder), feeding habits (whether the fish is herbivorous, carnivorous, or omnivorous), the type of pollutants present in their aquatic environment, fish size, metabolic rate, and biotransformation ability^11^. For instance, freshwater fish such as rainbow trout can absorb a high content of iron from the water through their gills, whereas marine fishes rely entirely on their food, which is rich in iron, rather than utilizing a gill uptake system like freshwater fishes^34^. Therefore, iron was not detected in all three marine fishes of our present study. Additionally, small amounts of aluminum (Al), zinc (Zn), titanium (Ti), and sodium (Na) were detected in the three fish species under investigation. The concentration of metals in *Sardinella abella* followed the order Cu > Zn > K > Ca > Ti, with titanium being a newly discovered metal in *Sardinella abella* from the Gawadar coast. In *Otolithes ruber*, the trace metal concentration was observed as K > Cu > Na, with potassium (K) exhibiting the highest value among the metals analyzed. Moreover, Table 4 revealed that no trace metals were detected in sample A1 of *Rastrelliger kanagurta*, while significant metal traces were observed in other samples (samples B1 and C1) of the same species. Such variations in the accumulation of trace metals in fish samples, even within the same species, might be attributed to several possible reasons including i.e., fish samples collected from areas close to pollution sources may exhibit differences in metal concentrations. Additionally, individual fish within the same species may have varying metabolic rates or efficiencies in accumulating and eliminating metals from their bodies, leading to disparities in metal concentrations. Moreover, metals in fish tissues can interact with other organic substances, such as protein, which can affect their detectability through analytical methods. Numerous studies^7,12^ have revealed that when fish are exposed to elevated waterborne metals, they can absorb or ingest contaminated water and food and then bioaccumulate these metals through their gills, skin and digestive tract. Consequently, the levels of metal concentration in each fish sample are mostly associated with sediment composition, changes in water quality, or the amount of contaminated water or food ingested. All these factors contribute to the variability observed in metal detection in fish samples, even within the same fish species.

Furthermore, the concentration of trace metals such as Na, Zn, Cu, K, Ca, Al and Ti observed in three fish species of our present study were also differs from the findings of previous workers^35–44^, who reported these metals in same fish species collected from the Pakistan, India, Oman and Nigerian coastal areas as presented in Table 12, respectively. Such comparison of trace metal concentrations in these fish species from different studies provides valuable insights into the potential variations in metal accumulation patterns among fish populations in the different locations. Understanding these variations is crucial for assessing the potential health risks associated with metal contamination and implementing appropriate monitoring and mitigation strategies. Chatterjee et al.^45^ reported that the presence of copper and zinc in fish is regulated through metabolic activities. Calcium, zinc, sodium, potassium, iron, and copper are essential minerals that play crucial roles in the health and well-being of fish. Calcium is a it is a major component of its bones and scales, and plays a crucial role in maintaining the structural integrity of the skeletal system. Zinc is essential for wound healing, reproduction, and maintaining a healthy immune system of fishes. Sodium in fish bodies can ensure the proper cell function, nerve transmission, and osmotic balance. Potassium is involved in nerve impulse transmission, muscle contraction, and the regulation of pH in fish. Iron is necessary for oxygen transport, growth and metabolism in fish. Its deficiency can lead to anemia diseases. Copper is a trace element that serves as a cofactor for several enzymes involved in important physiological processes, such as respiration and immune functions. Although, these metals in small amounts are essential for the regulation of metabolic activities of fish body, but their imbalance or deficiency of any of them can lead to various health issues in fishes^46^. Changes in mineral content in fish muscles are primarily influenced by the rate at which the fish can absorb inorganic materials from the surrounding water body^47^. Accumulation of these metals in fish is not solely dependent on water; other abiotic factors in the aquatic environment and the duration of fish exposure to these factors also play a significant role in metal bioaccumulation. The concentration of trace metals in fishes varies due to environmental conditions, seasonal changes, temperature, salinity, sexual maturity, age, habitat, and feeding habits, as observed by Yildiz^48^. Such variations can also occur among different body parts of the same fish, such as muscle tissue and liver tissue, and are influenced by the age of the species^10^.

**Table 12:** Comparison of mean concentration of studied trace metal levels in three fish species of Gwadar coast with previous related literatures.

Metals	Fish species	Average measured values of metals in studied fish species in mg/kg	Previous published Trace metal concentration in studied fish species	fish samples	location	References
Al	*R. kanagurta*	0.66	34.66–58.55 μg/g	Muscles	Visakhapatnam coast, India	Mangalagiri et al.^35^
	*S. abella*	ND	NA			
	*O. rubber*	ND	0.67–0.84 mg/kg	Processing discrad of fish body region	Local fish market, Kerala, India	Renuka et al.^36^
K	*R. kanagurta*	2.675	2397 mg/100 g w.w	Muscles	Indian coast	Mohanty et al.^37^
	*S. abella*	1.94	1.33 mg/kg	Whole fish	Minnna metropolis, Niger State of Nigeria	Ako and Salihu^38^
	*O. rubber*	0.00061	30.97–83.57 mg/kg	Processing Discrad of fish body region	Local fish market, Kerala, India	Renuka et al.^39^
Cu	*R. kanagurta*	12.71	2.03–8.21 µg/g;	Muscles	Karachi coast, Pakistan;	Al-Shwafi,^40^; Ahmed et al.^41^
			0.11 ± 0.03 ppm;		Red Sea of Yemen;	
			0.09 ± 0.04 ppm		Gulf of Aden	
	*S. abella*	4.14	1.69 µg/g	Muscles	Gwadar coast, Pakistan	Ahmed et al.^42^
	*O. rubber*	0.0053	0.07–0.14 mg/kg	Processing Discrad of fish body region	Local fish market, Kerala, India	Renuka et al.^36^
Zn	*R. kanagurta*	6.64	2.96–9.41 µg/g	Muscles	Karachi coast, Pakistan;	Al-Shwafi,^40^; Ahmed et al.^41^
			0.75 ppm		Red Sea of Yemen;	
			0.55 ppm		Gulf of Aden	
	*S. abella*	2.96	1.88 µg/g	Muscles	Gwadar coast, Pakistan	Ahmed et al.^42^
	*O. rubber*	ND	2.4–8.5 µg/g	Muscles	Pozm Bay, North of Oman Sea	Balooch et al.^43^
Ca	*R. kanagurta*	ND	429 mg/100 g	Whole fish	Indian Coast	Gopalan et al.^44^
	*S. abella*	0.77	1.61 mg/kg	Whole fish	Minnna metropolis, Niger State of Nigeria	Ako and Salihu^38^
	*O. rubber*	ND	9.73–37.0 mg/kg	Processing discrad of fish body region	Local fish market, Kerala, India	Renuka et al.^36^
Ti	*R. kanagurta*	ND	NA			
	*S. abella*	0.45	NA			
	*O. rubber*	ND	NA			
Na	*R. kanagurta*	ND	107 mg/100 g w.w	Muscles	Indian coast	Mohanty et al.^37^
	*S. abella*	ND	1.58 mg/kg	Whole fish	Minnna metropolis, Niger State of Nigeria	Ako and Salihu^38^
	*O. rubber*	0.0026	30.2–62.55 mg/kg	Processing Discrad of fish body region	Local fish market, Kerala, India	Renuka et al.^36^
Fe	*R. kanagurta*	ND	18.92 ± 13.12; 56.17 ± 24.23 µg/g	Muscles	Karachi coast, Pakistan	Ahmed et al.^41^
	*S. abella*	ND	3.82 µg/g	Muscles	Gwadar coast, Pakistan	Ahmed et al.^42^
	*O. rubber*	ND	01.18–2.26 mg/kg	Processing discrad of fish body region	Local fish market, Kerala, India	Renuka et al.^36^

*ND* not determined, *NA* Not available, *w.w* wet weight.

### Human health risk assessments

Human health risk assessments, according to USEPA^25^, can be defined as a process to estimate the nature and all possible adverse effects on human health when exposed to certain amounts of toxic chemicals that contaminate the environment. Therefore, to estimate the potential human health risk caused by chemical contaminants such as toxic metals, parameters suggested by the United States Environmental Protection Agency (EPA) were also utilized in this study. These parameters include the estimated daily metal intake (EDMI), target hazard quotient (THQ), total target hazard quotient (TTHQ), and target cancer risk (CR). Our present study revealed that THQ and TTHQ values of all six trace elements in *Rastrelliger kanagurta*, *Sardinella abella* and *Otolith ruber* were less than one. Hence, it is suggested that these three fish species show no adverse human health effects upon consumption. Wang et al.^49^ observed health risk issues associated with trace metals, i.e., Cd, Cr, Cu, Ni, Pb, and Zn in twelve marine fish occurring at Liaodong Bay on the China coast. They found that the total target hazard quotient (TTHQ) values were greater than one (TTHQ > 1) in these fishes and concluded that Chinese people were susceptible to high health risks by their consumption, which is consistent with our present study. On the other hand, Adebiyi et al.^50^ studied the health risk associated with potentially toxic heavy metals like Fe, Cu, Cd, Cr, and Pd in crayfish (*Palaemon hastatus*) commonly consumed by children (with an average body weight of 16 kg) and adults (with an average body weight of 70 kg) in Nigeria. They observed that in both cases for children and adults, the values of estimated daily metal intakes (EDMI) were found to be less than the oral reference dose (RfD), and the target hazard quotient (THQ) values were below one. Thus, the daily intake of these metals by ingesting crayfish in both children and adults has no adverse effect on human health. Accordingly, the accumulation of metals in body tissues is dose-dependent and related to exposure duration, reflecting the levels of metals in the environment. Javed & Usmani^51^ studied the accumulation of heavy metals and human health risk assessment in both adult males (with an average body weight of 57 kg) and females (with an average body weight of 50 kg) via the consumption of freshwater fish (*Mastacembelus armatus*) collected from Kasimpur canal in India. They indicated that inhabitants, particularly adult females who consume these fishes, were at a higher health hazard risk associated with metal toxicity than adult males. Likewise, Vahter et al.^51^ observed gender-related differences in exposure and health risk caused by heavy metal toxicity (cadmium, nickel, lead, mercury, and arsenic) in women than men. They indicated that women are more affected by metal contaminants than men due to the time of exposure to metals and high gastrointestinal absorption at adult age, whereas males seem to be more sensitive to exposure to metals during early development. According to Javed & Usmani^51^, the potential health risk caused by these chemical contaminants depends not only on the intake amount of these metals but also on exposure frequency and duration, oral reference dose (RfD), and the average body weight of its consumers. Therefore, if the EDMI value of a heavy metal is less than the RfD, then the health risk will be minimal. However, if EDMI is greater than 1–5 times the RfD, the risk will be low. If EDMI exceeds 5–10 times the RfD, the risk will be moderate. Furthermore, if EDMI exceeds 10 times the RfD, then the risk will be high^48^. THQ is a ratio of the concentration of heavy metal content in the food item to its RfD, weighed by duration and frequency of exposure, intake amount, and body weight. Therefore, if THQ > 1, it indicates potential health risks associated with metals found in our food. But if the THQ value is less than one, then it indicates a no potential non-carcinogenic risk associated with these metals to the exposed population. Moreover, it was also noted that THQ is not only a measure of health risk but also reflects the level of concern. Additionally, most previously published work in the areas of environmental toxicology and professional health involved male subjects only^52^.

The accumulation of trace elements from both natural and anthropogenic sources in marine organisms through the aquatic food web can pose health risks to consumers. However, there is limited research on the concentrations of trace elements in three specific marine fish species from the Gwadar coast of Pakistan, which is the focus of our present study. We determined the concentrations of eight trace elements (Na, Zn, Fe, Cu, K, Ca, Al, and Ti) in these marine fish species and estimated the dietary exposure of these trace elements to humans, assessing potential health risks. The persistence, bioaccumulation, bio-magnification, and toxicity of these trace elements in the food chain can lead to serious health hazards in humans. Trace metals are essential for various biological functions in the human body, but excessive exposure or imbalances can pose health risks^8,13^. These potential health risks associated with some of the metals includes i.e., **(1) Sodium (Na)** excessive daily intake cause high blood pressure, which can increases the risk of various other cardiovascular diseases; **(2) Zinc (Zn)** is an essential trace element, but its excessive intake can lead to nausea, vomiting, loss of appetite, and impaired immune function; **(3) Iron (Fe)** is crucial for trace metal for transporting oxygen in the blood, but excessive iron can lead to iron overload disorders that cause organ damage and other health issues; (4) **Copper (Cu)** is essential for various enzymes, but excessive copper intake can lead to gastrointestinal symptoms, liver damage, and neurological issues; **(5) Potassium (K)** is vital for nerve and muscle function, but abnormal levels can cause heart rhythm disturbances and other complications; **(6) Calcium (Ca)** is vital for nerve and muscle function, but abnormal levels can cause heart rhythm disturbances and other complications; (7) high exposure of **Aluminum (Al)** has been found to be associated with neurological disorders, such as Alzheimer's disease; **(8) Titanium (Ti)** is generally considered biocompatible and safe for use in medical implants^36^. However, inhalation of fine titanium particles may pose respiratory risks in certain occupational settings. Titanium was also studied by the World Health Organization in 1982 and accordingly, its intramuscular injection can induce both reproductive organ fibrosis and lymphosarcomas in rats. However, it was concluded that the available data from WHO on the carcinogenicity of titanium did not indicate such an effect on the human population. While 1500 µg/g/day was consider a non-toxic level for humans. Moreover, the health effects of these metals depend on various factors, including the usage of the metal, the route of exposure (ingestion, inhalation, or skin contact), and individual susceptibility. In many cases, the levels of these metals are tightly regulated by the body, and their deficiencies or excesses are relatively rare under normal dietary conditions. The contamination of seafood with trace metals is a global crisis, as seawater is increasingly susceptible to pollutants discharged along nearly every coast worldwide. If hazardous trace metals contaminate marine fishes and are consumed at a population level, human organs such as the liver, kidney, central nervous system, mucous tissue, intestinal tract, and reproductive system may accumulate these metals, causing severe damage. Therefore, understanding and addressing the trace metal contamination of seafood is crucial for safeguarding human health, especially considering the widespread discharge of pollutants into marine environments globally^53^.

Although fish body muscles are often measured with the slightest of heavy metal. Muscles usually are paid attention to because they are utilized in the human diet. Calcium according to prior research is a plentiful mineral that is required by the human body for normal growth and repair in muscles and the central nervous system (CNS). Calcium also plays a significant role in the blood clotting phenomenon and is stored in bones and teeth. Fewer levels of calcium may cause calcium rickets and osteomalacia. Most enzymes need zinc for their proper functioning plus these enzymes participate in anabolism and catabolism pathways Copper is a remarkable cofactor that has importance as a metabolite and aids in hemoglobin synthesis. All these reported trace metals of this study have significant importance because these are requirements for normal human body functioning that is crucial for life. Aluminum is our recent finding that needs more insight into it is required according to WHO limits. Thus, the comparison of trace metals in fish species from various studies highlights the complexity and multifactorial nature of metal accumulation processes. Factors such as environmental conditions, biological factors, and the physiological characteristics of fish species can all contribute to the observed variations. Understanding these factors is essential for assessing the potential risks associated with metal contamination in fish and developing appropriate management strategies to ensure the safety of fish consumption. Thus, our study will provide important information for individuals to make informed decisions about their diets, highlighting the significance of sustainable fishing practices to preserve these valuable marine resources. Overall, this research underscores the nutritional value of these commercially important marine fish species from the Gwadar Coast and emphasizes their role in contributing to a healthy diet.

## Conclusions

In conclusion, this study highlights the high protein content and slight variations in crude protein values among different fish species. These findings are consistent with previous research and indicate that fish can serve as a valuable protein source for human consumption. The observed variations in protein content can be attributed to factors such as environmental changes, growth stages, and seasonal fluctuations. Furthermore, the study reveals slight variations in lipid content among the analyzed fish species, influenced by environmental and biological factors affecting lipid metabolism and accumulation.

Regarding metal concentrations, the study findings differ from previous research, emphasizing the complexity of metal accumulation processes in fish. Understanding the potential health risks associated with metal contamination is crucial, and monitoring efforts should be implemented to assess variations in metal accumulation patterns among fish populations in different locations. By comprehending and managing these factors effectively, it is possible to ensure the safety and sustainability of fish as a nutritious food source while minimizing potential health risks for consumers.

## Data Availability

All data analyzed during this research project had been incorporated into this manuscript.
